# Changes in serum virus-specific IgM/IgG antibody in asymptomatic and discharged patients with reoccurring positive COVID-19 nucleic acid test (RPNAT)

**DOI:** 10.1080/07853890.2020.1811887

**Published:** 2020-10-01

**Authors:** Junli Liu, Rui Lian, Guochao Zhang, Baojun Hou, Chuming Wang, Jian Dong, Liu Yang, Jianglan Wang, Shangming Dai, Libo Chen, Guoqiang Zhang, Xin Lu, Ting Ye

**Affiliations:** a Laboratory Department, Union Jiangbei Hospital, Wuhan, China; b Emergency Department, China-Japan Friendship Hospital, Beijing, China; c General Surgery Department, China-Japan Friendship Hospital, Beijing, China; d Endocrinology Department, Union Jiangbei Hospital, Wuhan, China; e Respiratory Department, Union Jiangbei Hospital, Wuhan, China; f Emergency Department, Union Jiangbei Hospital, Wuhan, China; g Rheumatology and Immunology Department, Friendship Hospital, Beijing, China; h Wuhan First Bioscience Co, Ltd, Wuhan, China

**Keywords:** COVID-19, IgM/IgG antibody, asymptomatic patient, discharged patient with reoccurring positive nucleic acid test (RPNAT)

## Abstract

**Background:**

Studies have demonstrated the diagnostic efficiency of antibody testing in COVID-19 infection. There is limited data on the IgM/IgG changes in asymptomatic and discharged patients with reoccurring positive nucleic acid test (RPNAT). This study aims to investigate these IgM/IgG changes.

**Methods:**

There were 111 patients with positive nucleic acid test (NAT) and 40 suspected patients enrolled in the study. The serum SARS-CoV-2 specific IgM/IgG antibody levels were retrospectively analysed with the disease progress in asymptomatic and RPNAT patients.

**Results:**

The best overall performance was found by combining the IgM, IgG, and CT; 95.1% sensitivity and 75% specificity. This was tested in 111 RT-PCR positive cases. The median IgM and IgG levels were lower in the asymptomatic group compared to the symptomatic group (*p* < .01). Among 15 RPNAT cases, the IgM levels of the RPNAT group at the time of discharge (IgM2.79, IQR: 0.95–5.37) and retest (IgM 2.35, IQR: 0.88–8.65) were significantly higher than those of the non-reoccurring positive nucleic acid test group (Non-RPNAT) (IgM on discharge: 0.59, IQR: 0.33–1.22, IgG on retest: 0.92, IQR: 0.51–1.58).

**Conclusion:**

Serum SARS-CoV-2 specific IgM/IgG antibody levels remained at a low level during hospitalisation for asymptomatic patients. Elevated IgM levels may have implications in the identification of RPNAT patients before discharge.

**Key messages**
- This study determined the IgM/IgG changes in asymptomatic and RPNAT patients. The rate of serum SARS-CoV-2 specific IgM/IgG antibody levels increase in the asymptomatic group was lower than in the symptomatic group during hospitalisation. The IgM level did not decrease significantly at discharge in the RPNAT patients, and was higher than that of the Non-RPNAT group on discharge. These results highlight the importance of timely monitoring of IgM levels to identify RPNAT patients before discharge.

## Introduction

The outbreak of Coronavirus Disease 2019 (COVID-19) has created a serious global public health threat. The disease has spread rapidly to other countries. The disease has spread rapidly to other countries since the first case was detected in Wuhan, China in December 2019 [1]. The World Health Organisation (WHO) has declared the ongoing outbreak as a global public health emergency [2]. Early and rapid SARS-CoV-2 identification is essential for timely and appropriate quarantine, and clinical management [3–6]. Reports [7–9] indicate that the initial symptoms as well as abnormal computed tomography (CT) images are essential for screening infected cases. The virus nucleic acid real-time reverse transcriptase-polymerase chain reaction (RT-PCR) test is considered as the “gold standard” for diagnosis. Asymptomatic cases with no history of positive contact may be identified using RT-PCR or imaging. Since limited data is available for asymptomatic cases [10] and some investigators [11] estimate that nearly a half of COVID-19-infected individuals are asymptomatic, the impact of asymptomatic transmission on the epidemic potential of COVID-19 has caused great concern.

Many studies [8,9,12,13] have investigated the clinical, laboratory and radiological features of confirmed symptomatic COVID-19 patients. However, there is little attention given to the follow-up of recovered patients. A recent study [14] indicates that four medical professionals in China tested positive for COVID-19 post-recovery; they had met the criteria for hospital release. An increasing number of similar cases have also been reported in other provinces in China. A positive RT-PCR test suggests that the recovered patients may carry the virus, complicating the efforts to control the outbreak. The identification of discharged COVID-19 patients with reoccurring positive nucleic acid test (RPNAT) remains uncertain. Due to the limits of the “gold standard” RT-PCR test, correct utilisation and observation of the dynamic changes of the IgM/IgG antibody may be helpful in the management of asymptomatic and RPNAT patients [15]. The IgM/IgG diagnostic efficiency [16–17] varies and the dynamic trend is not well known. This study sought to determine whether the IgM/IgG levels differed between asymptomatic and symptomatic cases, and to evaluate the utility of IgM/IgG levels to distinguish between RPNAT patients and non-reoccurring positive nucleic acid test patients (Non-RPNAT).

## Materials and methods

### Patients and samples

This retrospective study was conducted in the Union Jiangbei Hospital, Wuhan, China. It is a 1100-bed tertiary teaching hospital that serves as one of the designated hospitals according to the government emergency rule of Hubei province. There were 111 patients with positive RT-PCR test results at the time of admission and 40 suspected patients from Feb 3 to Mar 13 were enrolled. The suspected cases were based on clinical manifestation, chest radiography and epidemiology. All suspected patients were eventually excluded based on clinical judgement as well as multiple negative RT-PCR tests. Data including epidemiological, clinical characteristics, laboratory results and imaging findings were uniformly collected. Paired nasopharyngeal swab and blood samples were taken from each patient. The study protocol was approved by the local ethics board (No. LLSC 2020032001). Consent was obtained from all the patients or their guardians.

### Definitions

A mild case was defined as a confirmed case with mild clinical symptoms without pneumonia imaging according to the 5th edition of COVID-19 Diagnosis Guidelines released by China’s National Health Commission [18]. A common case was defined as fever and/or other respiratory presentation with pneumonia under radiography. A severe case was defined as dyspnoea or respiratory failure including one of the following: (1) Dyspnoea, RR ≥ 30 times/minute; (2) finger oxygen saturation under resting ≤ 93%: and (3) arterial PaO2/FiO2 ≤ 300 mmHg. While an asymptomatic case in our study was defined as a PT-PCR test positive case with normal body temperature, no discomfort and without pneumonia imaging during hospitalisation.

Discharge standards must meet all of the following [18]: (1) The body temperature returned to normal >3 days; (2) Respiratory symptoms improved significantly; (3) Pulmonary imaging showed obvious absorption of acute exudative lesions; (4) RT-PCR test was negative for two consecutive respiratory tract samples (sampling time at least 24 h apart).

Discharged patients with reoccurring positive nucleic acid test (RPNAT): The nucleic acid retest turned positive after the patient was discharged from the hospital.

### Measurement of serum SARS-CoV-2 specific IgM/IgG antibody

Serum SARS-CoV-2 specific IgM-IgG antibody levels were measured using COVID-19 IgG Detection Kits (Magnetic Beads Chemiluminescent Immunoassay), which were purchased from Hunan Yuanjing Biotechnology Co., Ltd. The schematic diagram of the detection kit structure is shown in Figure S1. This assay is based upon the two-steps indirect method utilising the SARS-CoV-2 spike receptor-binding domain (S-RBD) and N spike protein as antigens. The detection process of chemiluminescence SARS-CoV-2 antibody reagent is shown in Figure S2. In the first step, the sample undergoes automatic dilution with the instrument and then combined with recombinant COVID-19 antigen-coated paramagnetic particles. They are incubated; the COVID-19 IgG or IgM antibody present in the sample binds to the antigen-coated on the paramagnetic particles. Unbound serum proteins are removed during the washing step. In the second step, the enzyme (alkaline phosphatase) conjugated mouse monoclonal anti-human IgG or IgM antibody is added to the reaction mixture and incubated. The COVID-19 IgG or IgM antibody captured to the solid phase reacts with mouse monoclonal anti-human IgG antibody within the enzyme conjugate. A complex is generated between the solid phase, the COVID-19 IgG or IgM antibody within the sample and mouse monoclonal anti-human IgG or IgM antibody within enzyme conjugate by immunological reactions. The luminescent substrate is added resulting in a luminescent signal after a second wash to remove unbound conjugate; the signal is proportional to the antibody in the sample. The test results in the sample are expressed in COI. The detection process takes 20 min. No cross-reactivity with other respiratory viruses and beta coronaviruses was detected, according to the manufacturer’s instruction. The antibody levels were redetermined using a SARS-CoV-2 antibody test from another provider (Zhengzhou Autobio Diagnostics Co. Ltd.). Intraclass correlation coefficient (ICC) analysis showed that the results of the two kits were consistent (IgM *R*^2^: 0.9758, IgG *R*^2^: 0.9678) (*p* < .0001). There were no significant differences in antibodies concentrations between 2 IgM/IgG Detection Kits.

**Figure 1.**
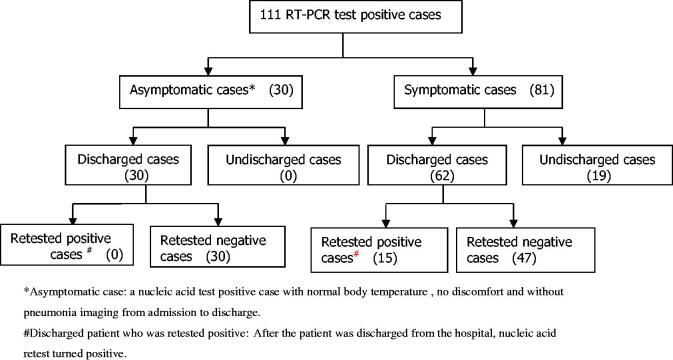
Patients flow and enrolment. *Asymptomatic case: a nucleic acid test positive case with normal body temperature , no discomfort and without pneumonia imaging from admission to discharge. #Discharged patient who was retested positive: After the patient was discharged from the hospital, nucleic acid retest turned positive.

**Figure 2.**
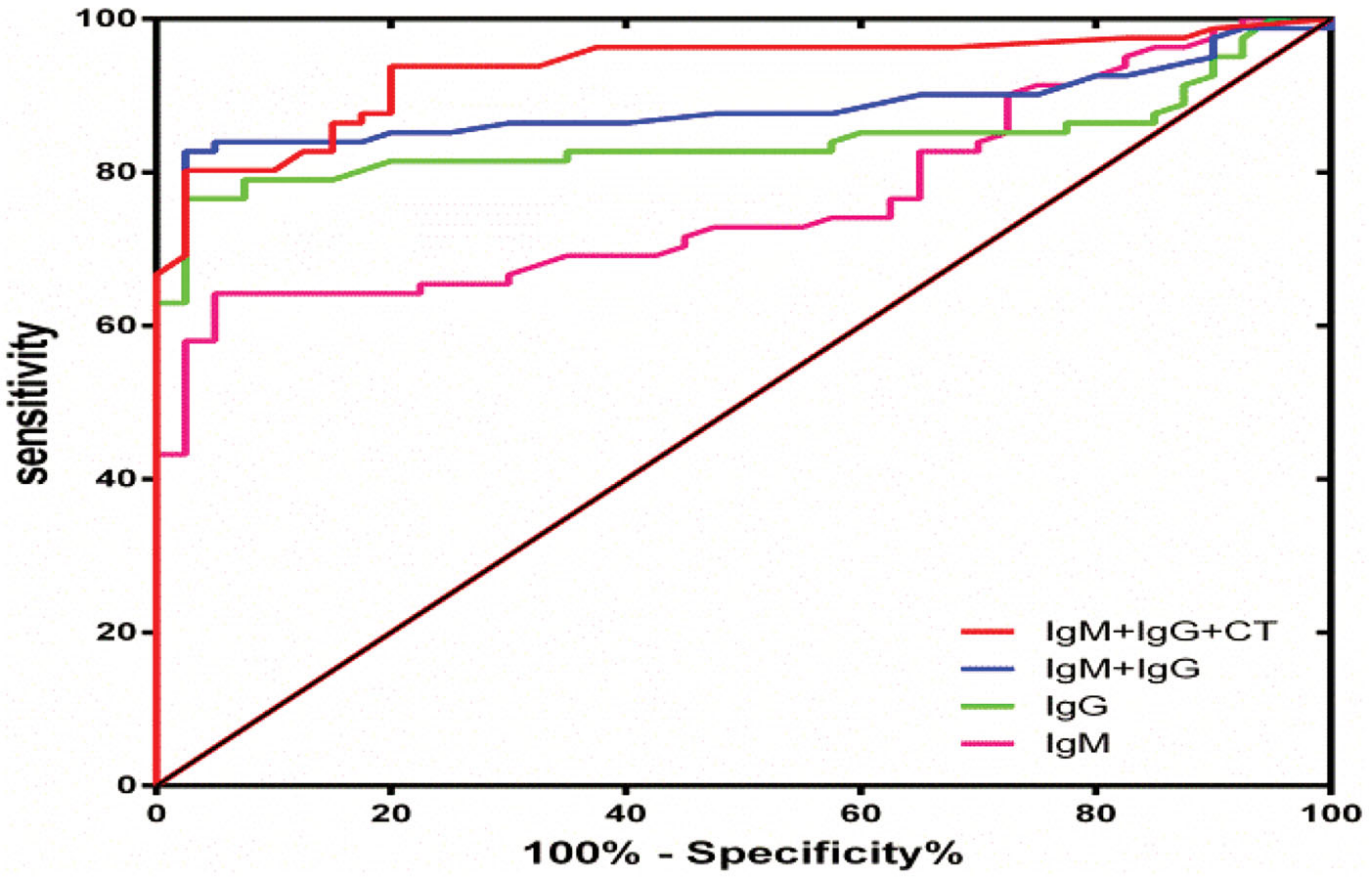
ROC curves of different testing items.

### Real-time reverse transcription polymerase chain reaction assay（RT-PCR）detection

The presence of SARS-CoV-2 in the respiratory specimens was detected using real-time RT-PCR amplification of SARS-CoV-2 open reading frame 1ab (ORF1ab), nucleocapsid protein (NP) genes fragments using kits (Shanghai BioGerm Biotechnology Co., Ltd). Conditions for amplification were 50 °C for 10 min, 95 °C for 5 min, followed by 40 cycles of 95 °C for 10 s and 55 °C for 40 s. The case would be considered to be laboratory-confirmed when two targets (ORF1ab, NP) tested positive using specific real-time RT-PCR [19]. A cycle threshold value (Ct-value) ≤ 38 was defined as a positive test, and a C_t_-value of >38 was defined as a negative test.

### Statistical analysis

Values were expressed as counts and percentages or median and inter-quartile range (IQR). Categorical variables were expressed as the number of cases. *T*-tests or Wilcoxon rank-sum tests were applied to continuous variables, whereas Fisher’s exact tests or chi-square tests were used for categorical variables. Corrected *p* < .05 values were considered statistically significant. Diagnostic accuracy was evaluated by sensitivity (SE), specificity (SP), positive and negative predictive value (PPV and NPV, respectively) for dichotomous tests and by the area (AUC) under ROC curve for quantitative tests. Data were analysed using GraphPad Prism 6.0 software (GraphPad Inc. San Diego, CA, USA).

## Results

### Patient characteristics

Patients’ demographic, clinical characteristics and laboratory results are shown in Table 1. Patients flow and enrolment is shown in Figure 1. Of the 111 RT-PCR positive cases, 17 (15.5%), 42(38.2%), 22 (20%) and 30(27.0%) were categorised into the severe, common, mild and asymptomatic groups, respectively. Blood samples were collected from the patients at various time-points after hospitalisation. The median time of the first blood collection in 81 symptomatic patients with initial symptoms was approximately 7 days (4, 14) after the symptom onset. The median time of the first blood collection was approximately 8 days (7, 9) in 30 asymptomatic patients after the positive RT-PCR test detection. The second blood samples collection was approximately 12 days (8, 18) after the onset of symptom or the positive RT-PCR test detection (A total of 65 blood samples were collected; blood samples were not collected from 16 patients because they were transferred to other hospitals). The RT-PCR test did not turn negative in 19 patients, while the rest of the patients turned negative and discharged. All 62 discharged patients were retested using nasopharyngeal swab; 15 turned positive RT-PCR results again, 54 of them (15 RPNAT patients, 39 Non-RPNAT patients) gave blood samples again in later check-ups.

**Table 1.** Demographic and clinical characteristics of patients.

Characteristics	COVID-19 patients (*n* = 81)	Asymptomatic cases (*n* = 30)	Suspected patients (*n* = 40)	*p* value
Age, Median (range), years	56 (23,93)	56.5 (20,94)	48.5 (23,98)	.274
Male, *n* (%)	48 (59.2%)	22 (73.3%)	23 (57.4%)	.330
Signs and symptoms, *n* (%)				
Fever (+)	57 (70.3%)	0	21 (52.5%)	.053
Other symptoms (+)	65 (80.2%)	0	27 (67.5%)	.122
Blood routine				
Leucocytes (× 10^9^ per L; normal range 4–10)	5.16 (4.15,6.39)	6.22 (5.43,7.42)	5.81 (4.88,6.86)	.005
Neutrophils (× 10^9^ per L; normal range 2–7)	2.95 (2.48,3.87)	4.09 (3.08,4.74)	3.64 (2.72,4.65)	.008
Lymphocytes (× 10^9^ per L; normal range 0.8–4)	1.47 (1.10,2.07)	1.86 (1.41,2.54)	1.81 (1.03,2.28)	.259
RT-PCR test (+), n (%)	81 (100%)	30 (100%)	0	<.001
CT (+),n (%)	66 (81.4%)	0	8 (20.0%)	<.001
Time from onset to blood samples collection (days)	7 (4,14)	8 (7,9)	9.5 (5,12)	.773
Outcome, *n* (%)				
Discharge	62 (76.5%)	30 (100%)	32 (80.0%)	.005
Hospitalisation	19 (23.5%)	0	8 (20.0%)	.005
Death	0	0	0	1.0

Other symptoms: dry cough, fatigue, dyspnoea, stuffy nose, sore throat, myalgia, diarrhoea and so on. CT: Computed Tomography. CT abnormality: Ground-glass opacities, consolidation, or both affecting at least one lobe. All suspected patients were eventually excluded from diagnosis due to comprehensive clinical judgement as well as multiple negative RT-PCR test.

### The diagnostic efficiency of serum SARS-CoV-2 specific IgM, IgG or combined with CT image

The interval between the occurrence of symptoms and the collection of blood samples is very important for this test. The first blood test time was at 7 days (4, 14) post infection. Symptoms appeared in 81symptomatic patients. Blood samples of 40 suspected patients were taken for control (blood sampling time 9.5(5, 12) days after the symptom onset). Among the 40 suspected patients, 2 patients had serum SARS-CoV-2 specific IgM only positive and 2 patients had IgG only positive. The IgM levels of these 4 patients were in the range of 0.54–3.27, while IgG 0.2–2.23. The rest of the suspected patients were serum SARS-CoV-2 specific IgM/IgG both negative. Of this 81 blood samples from SARS-CoV-2-infected patients and 40 blood samples from non-COVID-19 patients, we presented the sensitivity, specificity, PPV, NPV, LR+, LR– and AUROC values for IgM, IgG, IgM/IgG (either one of them positive), IgM/IgG/CT (either one of them positive) at different time point separately in Table S1 and Figure 2.

### Comparison of serum SARS-CoV-2 specific IgM/IgG levels between asymptomatic and symptomatic covid-19 patients

**Figure 3.**
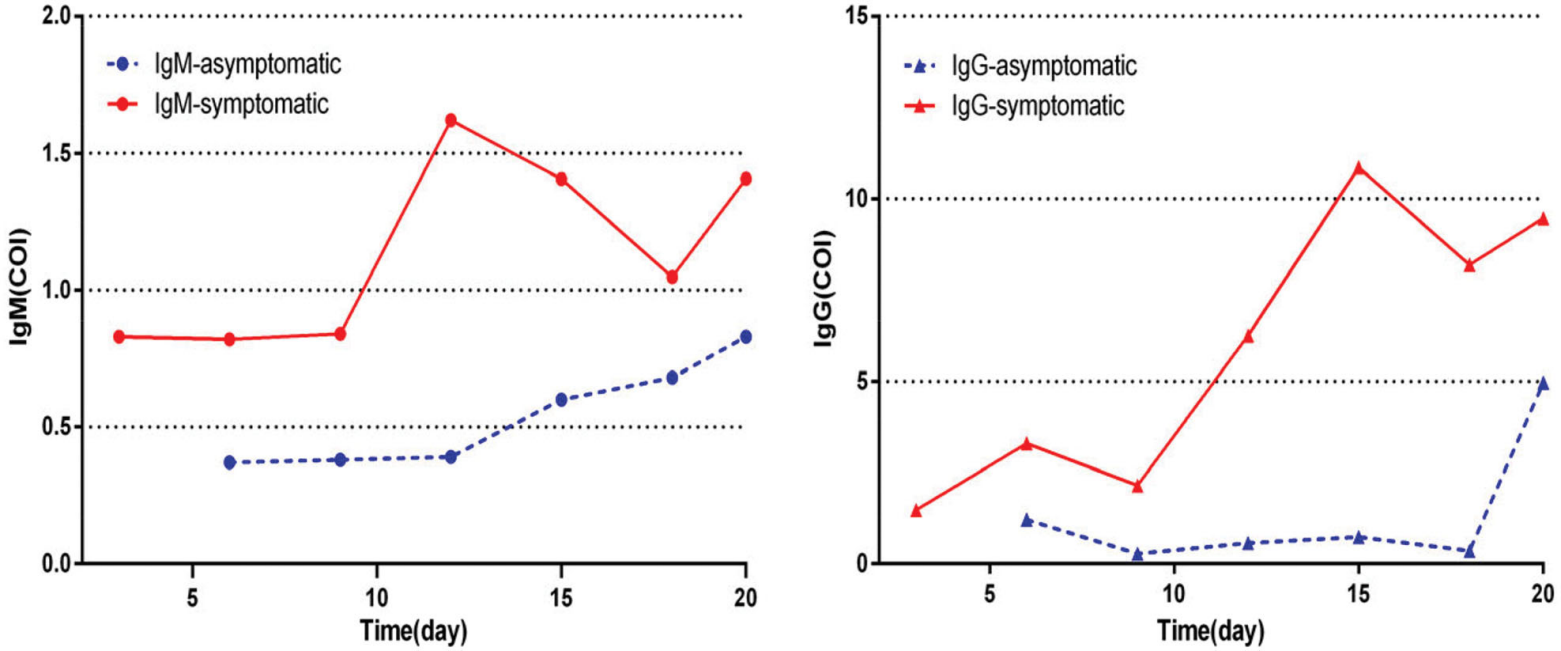
Dynamic changes of IgM/IgG levels in asymptomatic and symptomatic group. *Time point of blood collection for asymptomatic patients: from the day of close contact with the confirmed patient to blood samples collection. #Time point of blood collection for symptomatic patients: time from symptom onset to blood samples collection

There were 30 asymptomatic cases identified as positive RT-PCR test. The asymptomatic cases (22 male and 8 female) aged between 20 and 94 years old (median age 55.1 years old). Among the asymptomatic patients, 17 (56.7%) were negative for both serum SARS-CoV-2 specific IgM and IgG antibody test. Therefore, the antibody levels of asymptomatic cases and symptomatic Covid-19 patients were compared and analysed (Table 2). The median serum SARS-CoV-2 specific IgM and IgG levels were lower for asymptomatic patients (IgM0.37, IQR: 0.24–0.78, IgG0.38, IQR: 0.17–1.45) compared to symptomatic patients (IgM1.73, IQR: 0.56–3.74, IgG5.67, IQR: 0.79–18.5) (*p* < .01) at the early stage of the disease. The median serum SARS-CoV-2 specific IgM and IgG levels in the asymptomatic group before and after RT-PCR turned negative are shown in Table 3. Both IgM and IgG levels remained low during hospitalisation (Figure 3). Documents on epidemiological investigation and medical records were retrospectively reviewed to demonstrate possible transmission potential of asymptomatic cases; these asymptomatic patients were not tracked. All asymptomatic cases were tested negative after discharge.

**Table 2.** Comparison of IgM/IgG levels at different time points between asymptomatic and symptomatic COVID-19 patients.

	Asymptomatic patients (*n* = 30)	Symptomatic patients (*n* = 81)	*p* value
Male, *n* (%)	22 (73.35)	48 (59.2)	.172
Age, Median (range), years	56.5 (20,94)	56 (23,93)	.566
Time point of blood collection*	8 (7,9)	7 (4,14)	.452
IgM	0.37 (0.24,0.78)	1.80 (0.57,4.92)	<.001
IgG	0.38 (0.17,1.45)	6.73 (1.73,13.88)	<.001

*Time point of blood collection for asymptomatic patients: from the day of close contact with the confirmed patient to blood samples collection. Time point of blood collection for symptomatic patients: time from symptom onset to blood samples collection.

**Table 3.** Comparison of IgM/IgG levels in asymptomatic group before and after RT-PCR turned negative.

	RT-PCR (+)	RT-PCR (–)	*p* value
IgM	0.37（0.24,0.78）	0.67（0.37,0.91）	.105
IgG	0.38（0.17,1.45）	0.73（0.35,1.65）	.193

### Serum SARS-CoV-2 specific IgM/IgG levels in RPNAT and Non-RPNAT groups

All discharged symptomaticCOVID-19 patients were transferred to a designated medical unit for extra 14 days’ quarantine period. Fifteen discharged patients (16.3%) were positive for RT-PCR retest. Baseline clinical and laboratory characteristics in the RPNAT and Non-RPNAT groups at the time of retest were recorded (Table 4). The serum SARS-CoV-2 specific IgM/IgG levels of the two groups were compared at different time points (on admission, on discharge, and retest). There was no difference in serum SARS-CoV-2 specific IgM and IgG levels between the RPNAT and Non-RPNAT groups (IgM in RPNAT group: 4.33, IQR: 0.75–9.04, IgM in Non-RPNAT group: 1.96, IQR: 0.41–4.59) on admission. However, serum SARS-CoV-2 specific IgM levels in the RPNAT group at the time of discharge (IgM2.79, IQR: 0.95–5.37 and at retest: IgM 2.35, IQR: 0.88–8.65) were significantly higher than those of the Non-RPNAT group (IgM on discharge: 0.59, IQR: 0.33–1.22, IgM, whereas on retest: 0.92, IQR: 0.51–1.58). The RPNAT group also had a higher IgG level (IgG 17.23, IQR: 6.89–24.31) than the Non-RPNAT group (IgG 6.20, IQR: 1.19–11.48) at the time of retest. The increase in serum SARS-CoV-2 specific IgM and IgG levels correlated with the positive conversion of RT-PCR retests (Table 5).

**Table 4.** Comparison of clinical and laboratorial characteristics in RPNAT and Non-RPNAT group at the time of retest.

	RPNAT (*n* = 15)	Non-RPNAT (*n* = 39)	*p* value
Male, n(%)	11 (73.3%)	24 (61.5%)	.42
Age, Median (range), years	48 (34,77)	55 (28,89)	.374
Current smoker	2 (13.3%)	3 (7.7%)	.61
Time from onset to blood samples collection(days)	17 (15,18)	16 (12,20)	.713
Clinical classification			
Mild cases	5 (33.3%)	9 (23.1%)	.441
Common cases	9 (60.0%)	26 (66.6%)	.646
Severe cases	1 (6.6%)	4 (10.2%)	1.0
Chest CT	10 (66.6%)	15 (62.5%)	.79
Leucocytes (× 10^9^ per L; normal range 3.69–9.16)	5.53(4.17,7.05)	5.26 (4.04,6.79)	.499
Neutrophils (× 109 per L; normal range 2–7)	3.15(2.63,5.34)	2.91(2.06,3.98)	.297
Lymphocytes (× 109 per L; normal range 0.8–4)	1.41(1.1,2.08)	1.73(1.18,2.26)	.311

RPNAT: reoccurring positive nucleic acid test; Non-RPNAT: non-reoccurring positive nucleic acid test.

**Table 5.** Comparison of IgM/IgG levels in RPNAT and Non-RPNAT group at different time points.

	RPNAT (*n* = 15)	Non-RPNAT (*n* = 39)	*p* value
Time on admission			
IgM	4.33 (0.75,9.04)	1.96 (0.41,4.59)	.220
IgG	7.69 (3.65,10.26)	5.87 (0.79,18.87)	.511
Time on discharge			
IgM	2.79 (0.95,5.37)	0.59 (0.33,1.22)	.005
IgG	8.59 (3.36,10.36)	6.25 (2.14, 9.47)	.434
Time on retest			
IgM	2.35 (0.88,8.65)	0.92 (0.51,1.58)	.004
IgG	17.23 (6.89,24.31)	6.20 (1.92,11.48)	.005

## Discussion

The novel coronavirus (2019-nCoV) disease, first reported in Wuhan, China in December 2019, COVID-19, has spread rapidly around the world. Rapid detection of COVID-19 cases requires the availability of rapid as well as accurate diagnostic testing, severe illness. Efficient detection is key to appropriately quarantine infected patients and block the spread of the virus [20,21]. However, the symptoms of COVID-19 are atypical and similar to other common respiratory diseases. The current gold standard, RT-PCR test, also has many limitations [22]. Therefore, rapid IgM/IgG antibody detection is considered as a supplementary diagnostic method. In terms of humoral immune response to pathogens, specific proteins, such as S and N protein of SARS-CoV-2, could stimulate the immune system to elicit an antibody response. The IgM antibody emerges first, followed by the IgA antibody and IgG antibodies respectively [23]. When the IgG antibody appears, its concentration continues to increase, the IgM decreases until it disappears, and the IgA antibody is sustained for a long time. The IgM antibody could be detected in patients’ blood after 3–6 days whereas IgG could be detected 8 days after SARS infection [15,24]. COVID-19 belongs to the family of viruses that cause outbreaks of Middle Eastern Respiratory Syndrome (MERS) and Severe Acute Respiratory Syndrome (SARS). Therefore Li et al. [15] hypothesised that COVID-19’s antibody production process is similar and the detection of IgG and IgM antibodies against SARS-CoV-2 would be indicative of infection.

Many studies [25,26] suggested that CT is valuable in the diagnosis of COVID-19. Therefore serum SARS-CoV-2 specific IgM, IgG, and CT detection of COVID-19 were combined to maximise the overall diagnostic accuracy. None of these tests is sufficiently accurate to identify COVID-19 infection singly as each shows an imbalance between sensitivity and specificity characteristics (Table 2). An incremental sensitivity was obtained when serum SARS-CoV-2 specific IgM, IgG, and CT were combined with either one of the three positive (median blood sample collected 7 days post symptom onset): sensitivity from 62.3 and 77.8% for IgM and IgG, respectively, to 95.1% when 3 tests combined, NPV from 55.2 and 67.8%, respectively, to 88.2% when combined. These 3 combined methods take only 30 min compared with the RT-PCR nucleic acid test, which takes at least 5–6 h. Therefore, these combined detections are effective to ensure timely quarantine and mitigate early spread, from 1-week after onset.

In this study, 30 asymptomatic cases account for 27.3% of positive RT-PCR cases during the same followed-up period. The main source of asymptomatic cases is close contacts of confirmed COVID-19 cases. The definition of asymptomatic cases in this study means that the patients are asymptomatic throughout the whole course of the disease. Wang et al. [27] reported clinical features of 55 asymptomatic patients on admission. Unlike in our study, the majority of the cases in their study advanced to mild and ordinary COVID-19 during the hospital stay. Hu et al. [28] provided evidence for transmission from an asymptomatic infector to close contacts that led to severe COVID-19 pneumonia. During the epidemic, these asymptomatic cases with different definitions have become a matter of great concern. Many investigators [29,30] believe that asymptomatic patients can spread infections, so their management is challenging. The serum SARS-CoV-2 specific IgM/IgG antibody levels of asymptomatic and symptomatic COVID-19 cases is unclear. The present study is the first to present that the median IgM and IgG levels are lower in the asymptomatic compared to symptomatic cases at the early stage of the disease. The curves show that the increasing rate of serum SARS-CoV-2 specific IgM/IgG antibody levels in the asymptomatic group was lower than in the symptomatic group during hospitalisation. More than half of asymptomatic cases were negative for both serum SARS-CoV-2 specific IgM and IgG antibody test on admission. First, since IgM antibody decreases and disappears after 2 weeks, we offer two possible explanations for these observations. In the asymptomatic cases, the time and duration of infection are still unknown. Therefore, the IgM level reduced or were even undetectable at the time of blood collection. Secondly, the knowledge of immune responses in asymptomatic cases is limited. The immune response in asymptomatic individuals is most likely localised at mucus membranes which do not induce adequate antibodies to be detectable in sera. Therefore, we hypothesise that a higher virus-specific antibody response is elicited in symptomatic compared to asymptomatic cases. Additionally, these asymptomatic cases were not tracked to infect others. It may be more appropriate for asymptomatic cases to be termed as asymptomatic carriers based on epidemiological investigations.

The emergence of RPNAT patients has aroused wide public concern with the control of the epidemic in China. Studies have shown the existence and clinical features of RP patients [14,31], however little attention has been paid to the immunological factors associated with the RPNAT patients. The persistence and clearance pattern of viral RNA in COVID-19 patients is unclear. The duration of virus RNA detection may be related to the host’s cellular immunity. The occurrence of RPNAT cases involves many factors, and its mechanism needs further study. The possibilities of positive RT-PCR retest results mainly depends on the following situations [32,33]: Previous false-negative RT-PCR test, persistent virus replication, delayed viral clearance due to corticosteroid treatment, intermittent excretion of virus and detection of inactive virus RNA. This study demonstrated dynamic changes of IgM/IgG levels between the RPNAT and the Non-RPNAT groups. Baseline characteristics between these 2 groups were similar at the time of retest (Table 5), and therefore comparable. The IgM levels at the time of discharge and recheck were significantly higher in the RPNAT group (*p* < .05) despite a small sample size. In the RPNAT group, the serum SARS-CoV-2 specific IgM level did not decrease significantly at discharge and was still higher than that of the Non-RPNAT group, suggesting that the presence of serum SARS-CoV-2 specific IgM were not always accompanied by a positive RT-PCR test result. Tan. et al suggested that [34] lymphopenia is an effective indicator for hospitalisation in COVID-19 patients. In the current study, there was no significant difference in the lymphocyte count in the two groups after discharge, all of which increased to >1000, so it is not reliable to judge virus clearance merely by the number of lymphocytes. Elevated serum SARS-CoV-2 specific IgM levels in RPNAT cases could indicate that the virus was replicating, implying that timely monitoring serum SARS-CoV-2 specific IgM levels is crucial especially in the late-stage disease. Therefore, this study recommends combining the RT-PCR test and the dynamic trends of IgM levels rather than nucleic acid test alone to determine whether the disease is in remission.

There are several limitations to our study. First, the data were collected retrospectively from patients hospitalised in a single centre and the population sample is small. It is unclear whether the results can be generalised to other patients, thus the numbers of enrolled patients should be increased. Second, only preliminary data had been collected and many patients remained in the hospital at the time of this writing; it is better to determine the progress of IgM/IgG levels and the outcomes of all patients.

## Conclusion

This study established the diagnostic performance of IgM, IgG, and CT in patients with COVID-19 infection. From our data, none of them proved better. Combined detection parameters are a reasonable choice. Persistent low levels of serum SARS-CoV-2 specific IgM/IgG in asymptomatic cases may correlated with lower virus-specific antibody immune response. We also provided evidence indicating that elevated serum SARS-CoV-2 specific IgM levels can aid to identify RPNAT patients before discharge.

## Supplementary Material

Supplemental Material
