# Supplementary material for: Changes in serum virus-specific IgM/IgG antibody in asymptomatic and discharged patients with reoccurring positive COVID-19 nucleic acid test (RPNAT)
Source: Ann Med. 2020 Oct 1;53(1):34–42. doi: 10.1080/07853890.2020.1811887 (PMC7544917; doi:10.1080/07853890.2020.1811887)

**Table S1:** Test characteristics of IgM, IgG, IgM/IgG, IgM/IgG /CT

|  | Sens, %  (95 % CI) | Spec, %  (95 % CI) | PPV, %  (95 % CI) | NPV, %  (95 % CI) | AUROC  (95 % CI) |
| --- | --- | --- | --- | --- | --- |
| IgM | 63.0  (51.5,73.4 ) | 92.5  (79.6,98.4 ) | 94.4  (84.6,98.8 ) | 55.2  ( 42.6,67.4) | 0.763(0.6806 ,0.8462 ) |
| IgG | 77.8  ( 67.2,86.3) | 95.0  (83.1,99.4 ) | 96.9  ( 89.3,99.6) | 67.8  ( 54.0,79.7) | 0.84(0.7676 ,0.9130 ) |
| IgM+IgG | 85.2  ( 75.6,92.1) | 90.0  (76.3,97.2 ) | 94.5  (86.6,98.5 ) | 75.0  (60.4,86.4 ) | 0.883(0.8208 , 0.9455 ) |
| IgM+IgG+CT | 95.1  (87.8,98.6 ) | 75.0  (58.8,87.3 ) | 88.5  ( 79.9,94.3) | 88.2  ( 72.5,96.7) | 0.933( 0.8895 ,0.9775) |

NPV, negative predictive value; PPV, positive predictive value; AUROC, area under receiver-operating curve;

**Figure S1**: Schematic diagram of the detection kit structure


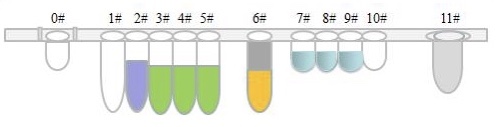


Description of each hole position :0#: Sample hole,1# :Reaction hole 2# :Sample diluent

3#4#5#: Cleaning fluid, 6#: YJAPS luminescent substrate solution ,7#: Test diluent ,8#: Magnetic particles coated with covid-19 antigen ,9#: Mouse anti-human IgG labeled AP ,10#: Spare hole ,11#: Reading hole

**Figure S2**: Detection process of chemiluminescence SARS-CoV-2 antibody reagent


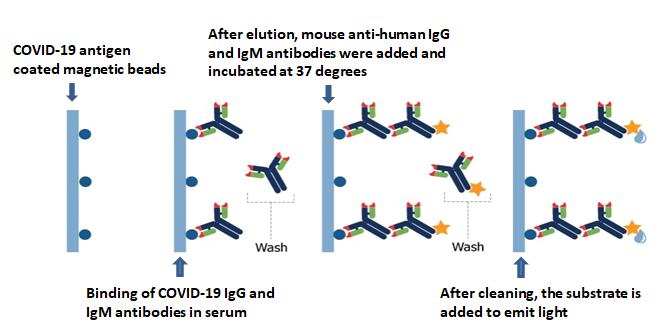

Supplement: Supplemental Material [file IANN_A_1811887_SM6749.docx]
